# Endoscopic ultrasound‐guided hepaticogastrostomy versus choledochoduodenostomy for malignant biliary obstruction: A meta‐analysis

**DOI:** 10.1002/deo2.274

**Published:** 2023-07-13

**Authors:** Hirofumi Yamazaki, Yasunobu Yamashita, Toshio Shimokawa, Kosuke Minaga, Takeshi Ogura, Masayuki Kitano

**Affiliations:** ^1^ Second Department of Internal Medicine Wakayama Medical University Wakayama Japan; ^2^ Clinical Study Support Center Wakayama Medical University Hospital Wakayama Japan; ^3^ Department of Gastroenterology and Hepatology Kindai University Faculty of Medicine Osaka Japan; ^4^ Second Department of Internal Medicine Osaka Medical and Pharmaceutical University Osaka Japan

**Keywords:** biliary obstruction, choledochoduodenostomy, EUS‐guided biliary drainage, hepaticogastrostomy, meta‐analysis

## Abstract

**Objectives:**

Endoscopic ultrasound (EUS)‐guided biliary drainage encompasses techniques such as EUS‐guided hepaticogastrostomy (EUS‐HGS) and EUS‐guided choledochoduodenostomy (EUS‐CDS). This meta‐analysis compared the efficacy of EUS‐CDS with that of EUS‐HGS for the treatment of biliary obstruction.

**Methods:**

A systematic meta‐analysis of all relevant articles listed was performed by searching the Cochrane Library, PubMed, and Google Scholar databases. We used random effects or fixed effects models to compare success rates, adverse events, procedure times, and time to recurrent biliary obstruction after EUS‐CDS and EUS‐HGS.

**Results:**

This meta‐analysis included 18 eligible studies. There was no significant difference between EUS‐CDS and EUS‐HGS with respect to technical success rate (odds ratio [OR] 1.04; 95% confidence interval [CI] 0.62–1.73) and clinical success rate (OR 0.66; 95% CI 0.43–1.04), or with respect to total procedure‐related adverse events (OR 1.39; 95% CI 1.00–1.93). Subgroup analysis of adverse events revealed that the rate of recurrent biliary obstruction (RBO) was significantly higher for EUS‐HGS (OR 2.95; 95% CI 1.54–5.64). There was no significant difference between the two methods with respect to time to recurrent biliary obstruction (mean difference –11.93 days; 95% CI –47.77–23.91). However, the procedure time was longer for EUS‐HGS (mean difference, 3.21 min; 95% CI 1.24–5.19).

**Conclusion:**

EUS‐CDS and EUS‐HGS are comparable in terms of technical success, clinical success, and rate of adverse events; however, EUS‐CDS is superior with respect to procedure time and preventing RBO.

## INTRODUCTION

Endoscopic retrograde cholangiopancreatography (ERCP) is the standard procedure for biliary decompression. However, ERCP results in difficult biliary cannulation in 15%–22% of patients,^1^, ^2^ and post‐ERCP pancreatitis in 3%–15% of patients.^3^ If ERCP fails, the choices are percutaneous biliary drainage (PTBD) or endoscopic ultrasound‐guided biliary drainage (EUS‐BD); however, PTBD requires an external drainage tube, it is invasive, and has a high risk of complications. Therefore, EUS‐BD is preferable. EUS‐BD encompasses techniques such as EUS‐guided hepaticogastrostomy (EUS‐HGS) and EUS‐guided choledochoduodenostomy (EUS‐CDS). Many reports have demonstrated the utility of EUS‐BD for patients with malignant biliary obstruction^4^, ^5^, ^6^, ^7^, ^8^, ^9^, ^10^, ^11^, ^12^, ^13^, ^14^, ^15^, ^16^, ^17^, ^18^, ^19^, ^20^, ^21^; however, whether EUS‐HGS is more effective than EUS‐CDS remains controversial. For that reason, we conducted a meta‐analysis to compare the usefulness and complication rates of EUS‐CDS with those of EUS‐HGS.

## METHODS AND LITERATURE SEARCH

This meta‐analysis did not require informed consent, ethical approval, or institutional review board approval. The review and meta‐analysis were reported and developed in accordance with the preferred reporting items for systematic reviews and meta‐analyses (PRISMA) guidelines.

From January 2001 to December 2021, an electronic search of the PubMed, Cochrane Library, and Google Scholar databases was performed. The search was limited to relevant English‐language articles and human studies, and used the following search terms: “biliary drainage”, “endoscopic ultrasound‐guided”, “hepaticogastrostomy”, “choledochoduodenostomy”, “stent patency”, “EUS‐guided biliary drainage”, “EUS‐guided hepaticogastrostomy”, or “EUS‐guided choledochoduodenostomy”. An expert methodologist (Toshio Shimokawa) supervised the systematic review and meta‐analysis. The reference list of each article was checked independently by two authors (Hirofumi Yamazaki and Yasunobu Yamashita) to identify any other relevant articles.

### Outcome measures

The primary outcome measures were the technical success rate, the clinical success rate, and the rate of adverse events (AEs). The secondary outcomes were procedure time and time to recurrent biliary obstruction (TRBO). The meta‐analysis of procedure time and TRBO was evaluated by calculating mean differences (MD).

### Outcome definitions

Technical success was defined as the suitable positioning of the stent, as determined by endoscopy and/or radiography. Clinical success was defined as a reduction in total serum bilirubin levels. AE was defined as procedure‐related events such as RBO, bleeding, cholangitis, pneumoperitoneum, bile leakage, and stent migration. RBO was defined as stent obstruction or stent migration. Procedure time was defined as the time from the puncture of the biliary tract by the EUS needle to the time of scope removal. TRBO was measured from the day of stent placement to the date of RBO.

### Inclusion and exclusion criteria

The inclusion criteria were as follows: full‐length articles written in English and comparing EUS‐HGS and EUS‐CDS with respect to technical success rate, clinical success rate, post‐procedure adverse events, procedure time, and/or TRBO. To assess TRBO, articles quoting a mean TRBO (and the standard deviation [SD]) were selected. Articles with incomplete data, case reports, reviews, conference abstracts, and editorials were excluded.

### Statistical analysis

Pooled odds ratios (ORs) and their 95% confidence intervals (CIs) were calculated based on the data reported in the collected studies. When there was no significant heterogeneity, the ORs for the outcome variables were calculated using a fixed effects model (Mantel‐Haenszel method).^22^, ^23^ If significant heterogeneity was present, a DerSimonian‐Laird random effects model was used. *I* ^2^ statistics of around 75%, 50%, and 25% were considered to indicate high‐, moderate‐, and low‐level heterogeneity.^24^, ^25^ Heterogeneity was evaluated using the Cochrane Q test and the *I* ^2^ statistic. The dispersion of data on the plot was assessed visually. Subgroup analyses were performed based on clinical factors such as AEs (RBO, bleeding, cholangitis, bile leakage, pneumoperitoneum, and stent migration). TRBO and procedure time were pooled with the MD. The SD of the mean TRBO was calculated. Potential publication bias was assessed using a funnel plot with Begg's rank correlation test and Egger's linear regression test.^26^, ^27^

A two‐sided *p*‐Value of <0.05 was considered statistically significant. Given the multiple comparisons, the results were interpreted carefully. All statistical analyses were performed by EZR statistical software (Easy R, Version 1.50; Jichi Medical University; Saitama, Japan).^28^ Two authors (Hirofumi Yamazaki and Yasunobu Yamashita) evaluated the Quality Assessment of Diagnostic Accuracy Studies‐2 (QUADAS‐2) to assess the quality of the chosen articles.^29^

## RESULTS

### Study selection and quality evaluation

After screening 6774 titles, 485 were excluded as duplicates, and a further 6246 were excluded after the title and abstract review. Full‐text review of the remaining 44 articles led to the removal of 26 studies that included no comparative data or were case reports. The remaining 18 studies were included in the final analysis; these 18 articles included 503 EUS‐HGS patients and 472 EUS‐CDS patients (Table 1). Fifteen of the cited studies included only patients with at least one failed ERCP. One study did not report primary drainage, one was a randomized study of ERCP or EUS‐BD, and the remaining study reported some patients that refused ERCP or failed ERCP. Overall, the percentage of patients with pancreatic cancer was 61.5% (506/823), although the rates in individual studies varied from 7% to 100%.

**TABLE 1:** Summary of the studies included in this meta‐analysis.

Author/year	Study type	Mean age, years (HGS/CDS)	Sample size (HGS/CDS)	Clinical success rate (HGS [%]/CDS [%])	Technical success rate (HGS [%]/CDS [%])	Adverse event rate (HGS [%]/CDS [%])	Type of stent
Cho et al. 2017	Prospective	66.3/64.0	21/33	85.7/100	100/100	38.1/30.3	PCSEMS
Park et al. 2011	Prospective	NR/NR	31/26	87.1/100	100/92.3	19.4/19.2	PS, FCSEMS
Kim et al. 2012	Retrospective	69.7/67.0	4/9	50.0/100	75.0/100	50.0/33.3	FCSEMS
Ardengh et al. 2018	Retrospective	68.2/73.5	6/6	83.3/83.3	66.7/88.3	16.7/0	FCSEMS
Khashab et al. 2016	Retrospective	63.6/67.6	61/60	82.0/85.0	91.8/93.3	19.7/13.3	MS, PS
Poincloux et al. 2015	Retrospective	NR/67	66/30	92.4/90.0	98.5/96.7	26.2/22.2	PS, FCSEMS, PCSEMS
Minaga et al. 2019	Prospective	72.5/73	24/23	87.5/95.7	87.5/82.6	25.0/17.4	PCSEMS
Guo et al. 2016	Retrospective	NR/NR	7/14	100/100	100/100	28.6/7.14	FCSEMS
Park et al. 2015	RCT	NR/NR	20/12	90.0/91.7	100/91.7	25.0/8.33	FCSEMS, PCSEMS
Artifon et al. 2015	RCT	66.3/65.8	25/24	88.0/70.8	96.0/91.7	20.0/12.5	PCSEMS
Ramírez‐Luna et al. 2011	Prospective	76.5/52.3	2/9	100/77.8	100/88.9	50.0/100	PS
Paik et al. 2018	RCT	NR/NR	32/32	81.3/87.5	96.9/91.0	NR/NR	PCSEMS
Sassatelli et al. 2019	Retrospective	66.3/73.5	20/13	75.0/76.9	100/100	20.0/0	PS, PCSEMS, LAMS
Kawakubo et al. 2014	Retrospective	72/72	20/44	NR/NR	95.0/95.5	30.0/13.6	PS, PCSEMS
Villa et al. 2012	Retrospective	NR/NR	34/26	NR/NR	64.7/73.1	32.4/15.4	NR
Amano et al. 2017	Prospective	75.1/72.5	9/11	NR/NR	100/100	11.1/18.2	PCSEMS, FCSEMS
Ogura et al. 2016	Retrospective	70/71	26/13	NR/NR	NR/NR	7.69/46.2	PCSEMS
Tyberg et al. 2018	Retrospective	69.9/69.7	95/87	NR/NR	91.2/92.0	21.1/29.9	PS, FCSEMS, PCSEMS, LAMS

Abbreviations: CDS, choledochoduodenostomy; FCSEMS, full covered self‐expandable metallic stent; HGS, hepaticogastrostomy; LAMS, lumen apposing metallic stent; NR, not reported; PCSEMS, partial covered self‐expandable metallic stent; PS, plastic stent.

**FIGURE 1:**
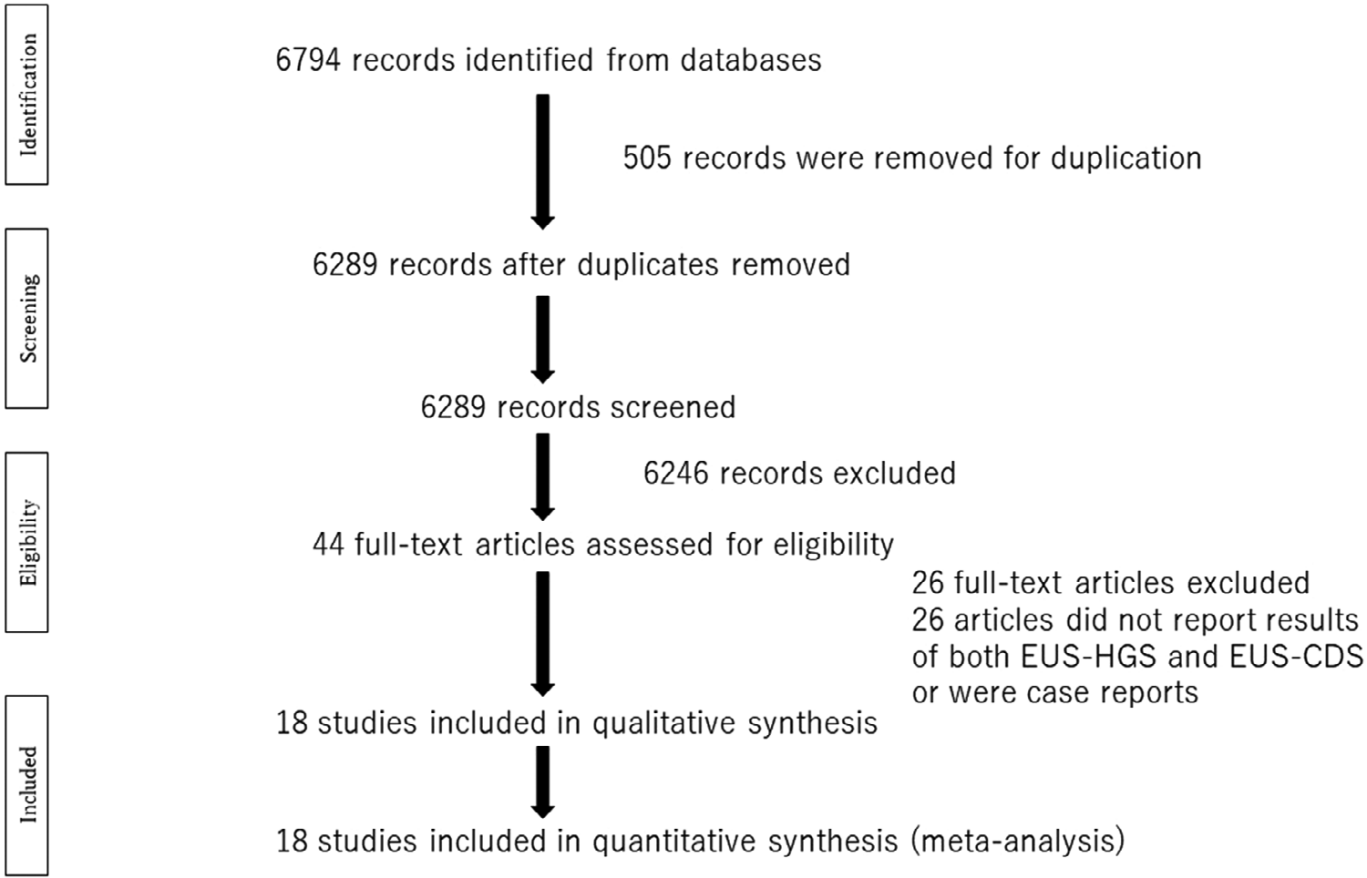
Flowchart illustrating the study selection process for the meta‐analysis. EUS‐HGS, endoscopic ultrasound‐guided hepaticogastrostomy; EUS‐CDS, endoscopic ultrasound‐guided choledochoduodenostomy.

**FIGURE 2:**
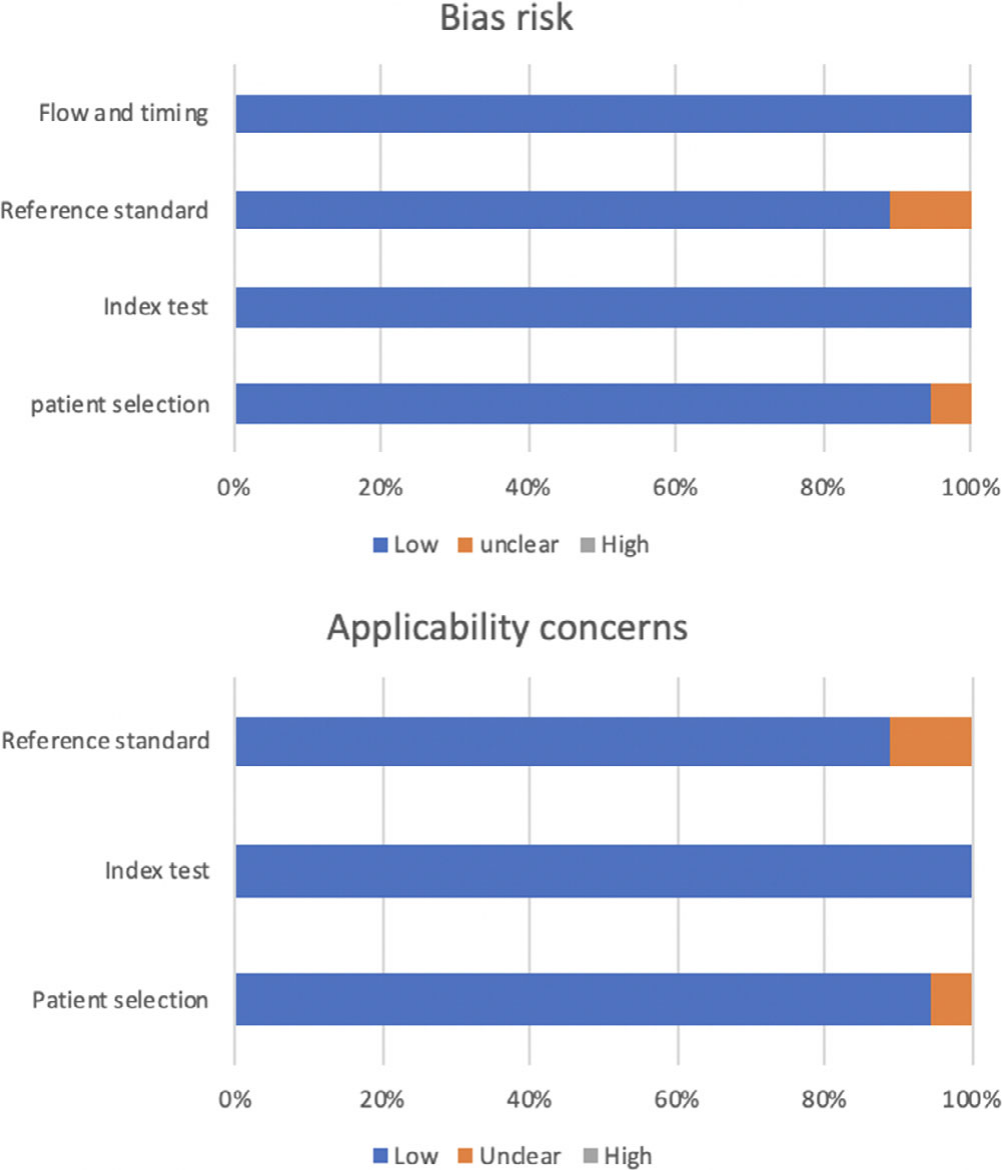
Quality assessment of studies according to QUADAS‐2. Gray = high risk of bias; Orange = unclear risk of bias; Blue = low risk of bias.

A flowchart illustrating the study selection process is shown in Figure 1. The main characteristics of the studies are listed in Table 1. Figure 2 and Table 2 show the QUADAS‐2 assessment.

**TABLE 2:** Quality assessment of studies according to QUADAS‐2.

	Risk bias				Applicability concerns		
Author/year	Patient selection	Index test	Reference standard	Flow and timing	Patient selection	Index test	Reference standard
Cho et al. 2017	Low	Low	Low	Low	Low	Low	Low
Park et al. 2011	Low	Low	Low	Low	Low	Low	Low
Kim et al. 2012	High	Low	Low	Low	High	Low	Low
Ardengh et al. 2018	Unclear	Low	Low	Low	Unclear	Low	Low
Khashab et al. 2016	High	Low	Low	Low	High	Low	Low
Poincloux et al. 2015	High	Low	Low	Low	High	Low	Low
Minaga et al. 2019	Low	Low	Low	Low	Low	High	Low
Guo et al. 2016	High	Low	Unclear	Low	High	Low	Unclear
Park et al. 2015	Low	Low	Low	Low	Low	Low	Low
Artifon et al. 2015	Low	Low	Low	Low	Low	Low	Low
Ramírez‐Luna et al. 2011	Low	Low	Low	Low	Low	Low	Low
Paik et al. 2018	Low	Low	Low	Low	Low	Low	Low
Sassatelli et al. 2019	High	Low	Low	Low	High	Low	Low
Kawakubo et al. 2014	High	Low	Unclear	Low	High	Low	Unclear
Villa et al. 2012	High	Low	Low	Low	High	Low	Low
Amano et al. 2017	Low	Low	Low	Low	Low	Low	Low
Ogura et al. 2016	High	Low	Low	Low	High	Low	Low
Tyberg et al. 2018	High	Low	Low	Low	High	Low	Low

### Primary outcomes

**FIGURE 3:**
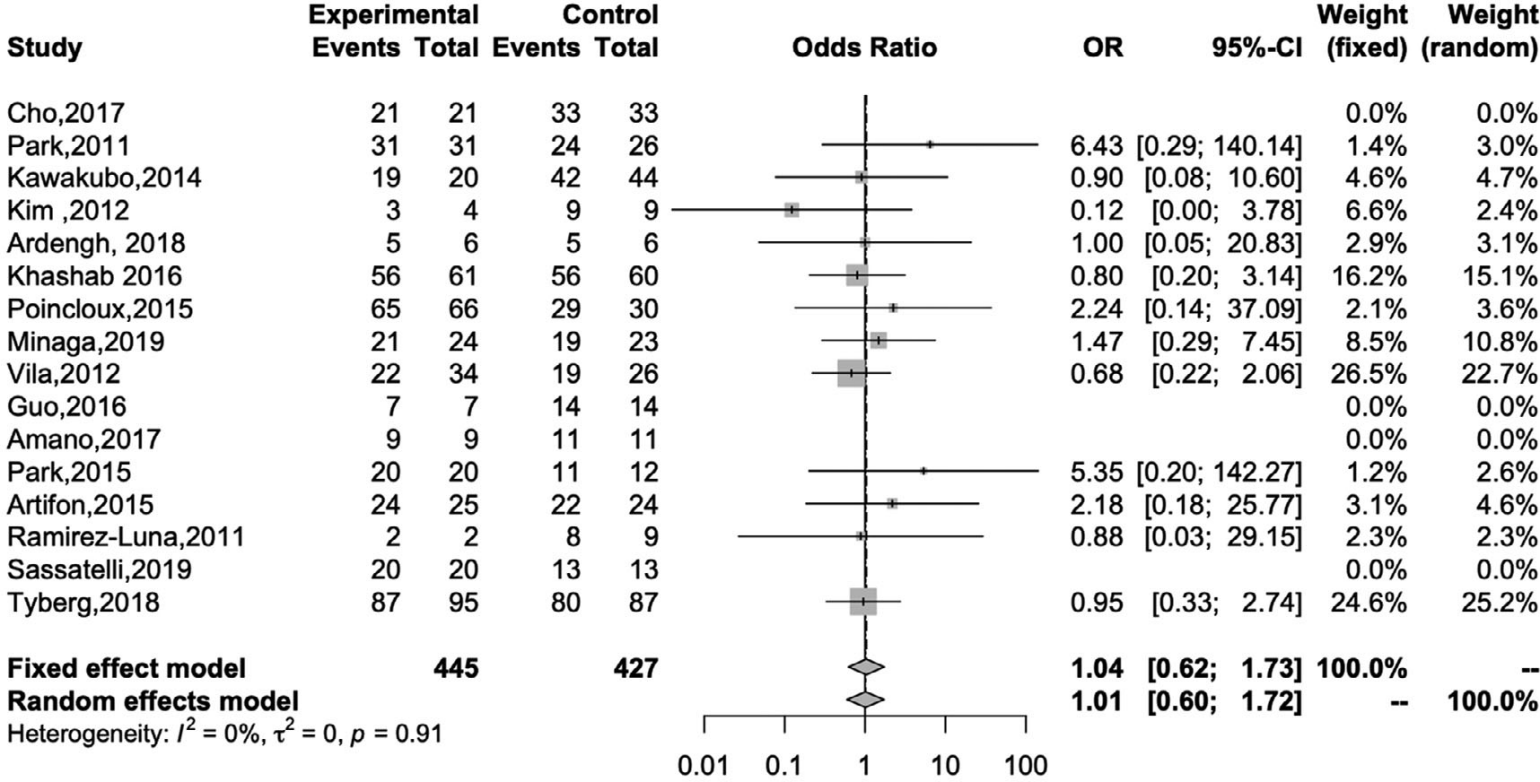
Forest plot comparing the technical success rates of EUS‐HGS and EUS‐CDS. OR, odds ratio; CI, confidence interval; EUS‐HGS, endoscopic ultrasound‐guided hepaticogastrostomy; EUS‐CDS, endoscopic ultrasound‐guided choledochoduodenostomy.

**FIGURE 4:**
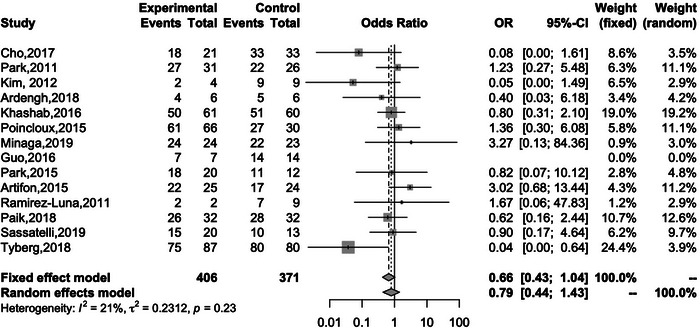
Forest plot comparing the clinical success rates of EUS‐HGS and EUS‐CDS. OR, odds ratio; CI, confidence interval; EUS‐HGS, endoscopic ultrasound‐guided hepaticogastrostomy; EUS‐CDS, endoscopic ultrasound‐guided choledochoduodenostomy.

Sixteen studies reported the technical success rates of both EUS‐HGS and EUS‐CDS. The overall technical success rates of EUS‐HGS and EUS‐CDS were 92.6% and 92.5%, respectively, and there was no significant difference between the two methods (OR 1.04; 95% CI 0.62–1.73; *I* ^2^ = 0%; *P*_heterogeneity_ = 0.91; Figure 3). Fourteen studies reported the clinical success of both EUS‐HGS and EUS‐CDS. The overall clinical success rates of EUS‐HGS and EUS‐CDS were 86.6% and 90.1%, respectively, again with no significant difference between the two methods (OR 0.66; 95% CI 0.43–1.04; *I* ^2^ = 21%; *P*_heterogeneity_ = 0.23; Figure 4).

**FIGURE 5:**
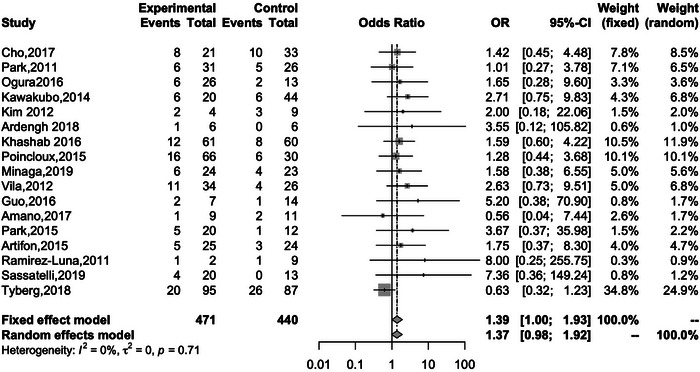
Forest plot comparing adverse events after EUS‐HGS and EUS‐CDS. OR, odds ratio; CI, confidence interval; EUS‐HGS, endoscopic ultrasound‐guided hepaticogastrostomy; EUS‐CDS, endoscopic ultrasound‐guided choledochoduodenostomy.

**FIGURE 6:**
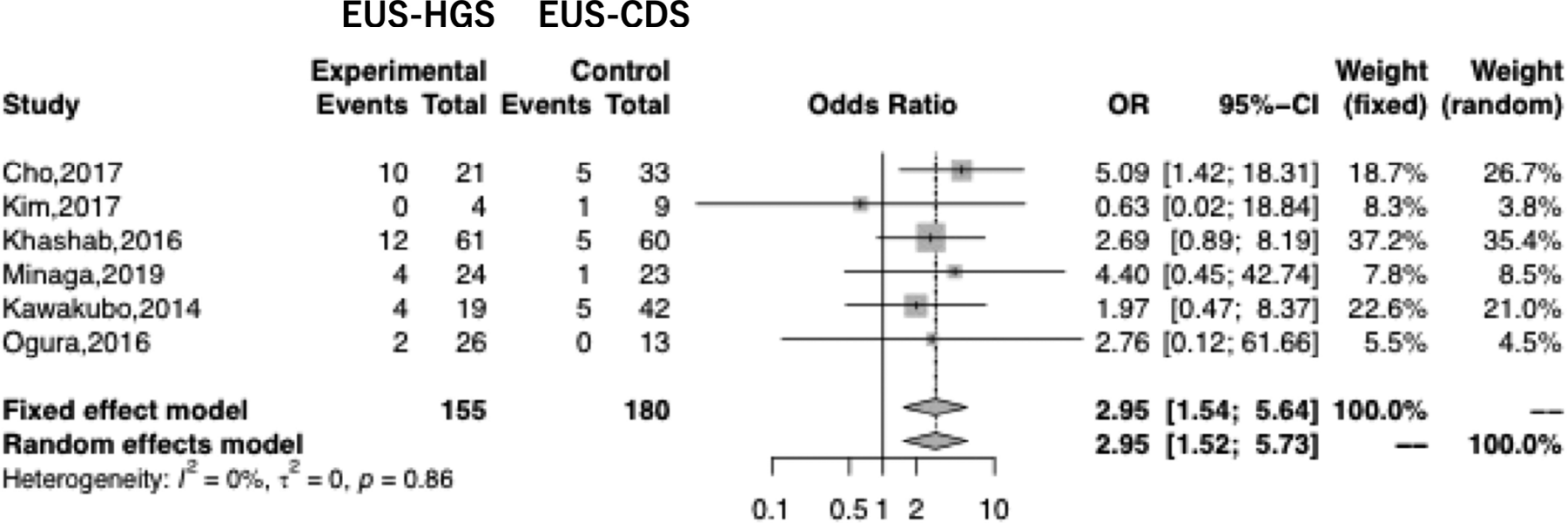
Forest plot comparing recurrent biliary obstruction (RBO) after EUS‐HGS and EUS‐CDS. MD, mean difference; CI, confidence interval; EUS‐HGS, endoscopic ultrasound‐guided hepaticogastrostomy; EUS‐CDS, endoscopic ultrasound‐guided choledochoduodenostomy.

Seventeen studies reported the rate of AE for both EUS‐HGS and EUS‐CDS. The overall rates for EUS‐HGS and EUS‐CDS were 23.8% and 18.6%, respectively, with no significant difference between the two methods (OR 1.39; 95% CI 1.00–1.93, *I* ^2^ = 0%; *P*_heterogeneity_ = 0.71; Figure 5). Subgroup analysis based on AE such as bleeding, cholangitis, RBO, bile leakage, pneumoperitoneum, and stent migration revealed a significant difference between EUS‐HGS and EUS‐CDS only with respect to RBO (Table 3). The overall rate of RBO for EUS‐HGS and EUS‐CDS was 20.6% and 9.44%, respectively (OR 2.95; 95% CI 1.54–5.64; *I* ^2^ = 0%; *P*_heterogeneity_ = 0.86; Figure 6). Concerning RBO, subgroup analysis was conducted based on stent type (partially covered self‐expandable metallic stent, PCSEMS), geographic region (Asia or not), and duodenal obstruction (occupying less than 50% of the patients in the study had duodenal obstruction). The results showed that the RBO rate for EUS‐CDS was significantly lower than that for EUS‐HGS. These results indicate that EUS‐CDS is superior to EUS‐HGS with respect to RBO (Table 4).

**TABLE 3:** Subgroup analysis of adverse events.

Outcome	OR (95% CI)	Heterogeneity, *I* ^2^
RBO	2.95 (1.54–5.64)	0%
Bleeding	0.46 (0.21–1.03)	0%
Cholangitis	1.59 (0.66–3.85)	10%
Bile leakage	0.72 (0.13–4.01)	51%
Pneumoperitoneum	0.74 (0.30–1.83)	29%
Stent migration	1.78 (0.50–6.36)	0%

Abbreviations: CI, confidence interval; OR, odds ratio; RBO, recurrent biliary obstruction.

**TABLE 4:** Subgroup analysis of factors related to recurrent biliary obstruction.

Outcome	OR (95% CI)	Heterogeneity, *I* ^2^
RBO (PCSEMS)	4.52 (1.56–13.11)	0%
RBO (duodenal obstruction <50%)	4.52 (1.56–13.11)	0%
RBO (Asian study)	3.09 (1.39–6.88)	0%

Abbreviation: CI, confidence interval; OR, odds ratio; PCSEMS, partial covered self‐expandable metallic stent; RBO, recurrent biliary obstruction.

### Secondary outcomes

**FIGURE 7:**
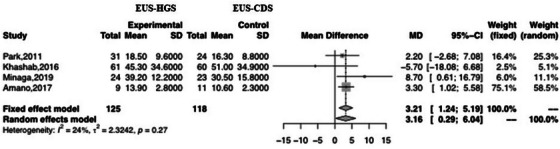
Forest plot comparing the procedure time of EUS‐HGS with that of EUS‐CDS. MD, mean difference; CI, confidence interval; EUS‐HGS, endoscopic ultrasound‐guided hepaticogastrostomy; EUS‐CDS, endoscopic ultrasound‐guided choledochoduodenostomy.

Four studies reported the time taken for EUS‐HGS and EUS‐CDS. There was a significant difference between the two, with the time for EUS‐CDS being shorter than that for EUS‐HGS (MD 3.21; 95% CI 1.24–5.19; *I* ^2^ = 24%; *P*_heterogeneity_ = 0.27; Figure 7).

**FIGURE 8:**
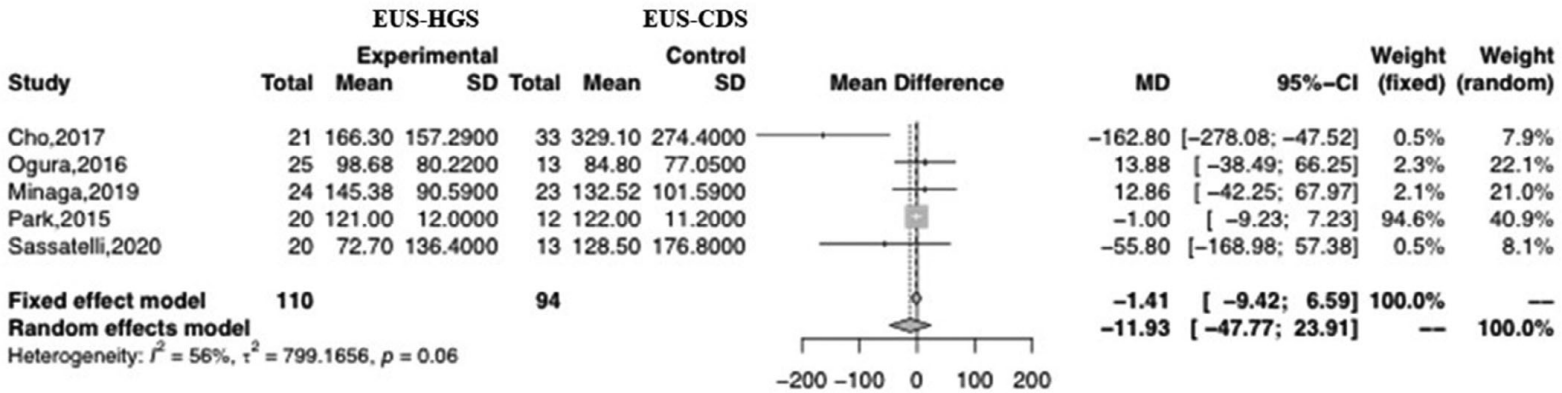
Forest plot comparing time to recurrent biliary obstruction after EUS‐HGS with that after EUS‐CDS. MD, mean difference; CI, confidence interval; EUS‐HGS, endoscopic ultrasound‐guided hepaticogastrostomy; EUS‐CDS, endoscopic ultrasound‐guided choledochoduodenostomy.

Of the 18 studies analyzed in this meta‐analysis, five were used to evaluate the mean TRBO. Only one reported sufficient data (mean ± SD) to calculate TRBO. Two studies estimated the mean and variance of TRBO (median, range, and 95% CI). The remaining two articles provided raw data (Table 5). There was no significant difference between EUS‐HGS and EUS‐CDS with respect to TRBO (MD –11.93; 95% CI –47.77–23.91; *I* ^2^ = 56%; *P*_heterogeneity_ = 0.06; Figure 8). Visual analysis of the plot revealed heterogeneity and high dispersion; therefore, we performed subgroup analyses to identify the underlying reasons. The subgroup analyses were based on stent type, geographic region (Asia or not), and duodenal obstruction (duodenal obstruction occupying less than 50%). However, the subgroup analyses revealed no significant differences between the groups, and heterogeneity remained (Table 6).

**TABLE 5:** Characteristics of the selected articles used to search for time to recurrent biliary obstruction.

	EUS‐CDS			EUS‐HGS		
Author/year	Sample size	TRBO, days (mean)	SD	Sample size	TRBO, days (mean)	SD
Cho et al. 2017	33	329.1	274.4	21	166.3	157.29
Ogura et al. 2016	13	84.8	77.05	25	98.68	80.22
Minaga et al. 2019	23	132.5	101.6	24	145.38	90.59
Paik et al. 2015	12	122	11.2	20	121	12
Sassatelli et al. 2019	13	128.5	176.8	20	72.7	136.4

Abbreviations: CDS, endoscopic ultrasound‐guided choledochoduodenostomy; EUS‐HGS, endoscopic ultrasound‐guided hepaticogastrostomy; SD, standard deviation; TRBO, time to recurrent biliary obstruction.

**TABLE 6:** Subgroup analysis of time to recurrent biliary obstruction.

Outcome	MD (95% CI)	Heterogeneity, *I* ^2^
TRBO (Asian study)	−1.14 (−47.70–30.17)	63%
TRBO (duodenal obstruction <50%)	−26.72 (−78.06–24.62)	65%
TRBO (PCSEMS)	−27.96 (−107.43–51.50)	75%

Abbreviations: MD, mean difference; PCSEMS, partial covered self‐expandable metallic stent; TRBO, time to recurrent biliary obstruction.

## DISCUSSION

When ERCP fails, EUS‐CDS and EUS‐HGS are suitable alternatives. Although previous meta‐analyses compared EUS‐HGS with EUS‐CDS,^30^, ^31^, ^32^ the meta‐analysis reported herein is, to the best of our knowledge, the largest to date. With respect to AE, there was a difference between EUS‐HGS and EUS‐CDS, but it was not significant. This result is similar to that of a previous meta‐analysis.^30^ AE such as cholangitis, bleeding, bile leakage, pneumoperitoneum, stent migration, and RBO can occur after EUS‐BD. This is the reason why we conducted subgroup analyses to examine the rates of RBO, bleeding, cholangitis, bile leakage, pneumoperitoneum, and stent migration after EUS‐HGS and EUS‐CDS. There was no significant difference between EUS‐CDS and EUS‐HGS in terms of rates of cholangitis, bile leakage, pneumoperitoneum, and stent migration. However, RBO was significantly less frequent after EUS‐CDS than after EUS‐HGS. This is consistent with the results of the most recent meta‐analysis.^30^ Subgroup analysis of the papers in which less than 50% of the patients in the study had duodenal obstruction also revealed a significant difference in RBO between EUS‐CDS and EUS‐HGS. This subgroup showed that the RBO rate of EUS‐HGS was higher than that of EUS‐CDS. However, subgroup analysis of papers in which more than 50% of patients in the study had duodenal obstruction was not possible due to the small number of papers (only two papers); one by Amano et al.^19^ and one by Ogura et al.^20^ However, Amano et al. did not report RBO, and Ogura et al. included only patients with duodenal obstruction. The latter found that EUS‐HGS may be better than EUS‐CDS for patients with duodenal obstruction as there were fewer AE. The results of this meta‐analysis and the study by Ogura et al.^20^ suggest that EUS‐CDS may be suitable for patients without duodenal obstruction; however, it remains controversial whether EUS‐HGS is better than EUS‐CDS for patients with duodenal obstruction.

TRBO did not differ significantly between EUS‐HGS and EUS‐CDS, although the heterogeneity for studies reporting TRBO was very high. Therefore, we performed a subgroup analysis to identify the underlying reason for this. Visual analysis of the resulting plot confirmed high heterogeneity. Cho et al.^4^ reported that EUS‐CDS has a longer TRBO than EUS‐HGS; however, two Japanese articles^10^, ^20^ reported that EUS‐HGS had a longer TRBO. When we conducted subgroup analyses (Asian population or not; the papers in which less than 50% of the patients in the study had duodenal obstruction), we found that these factors did not affect heterogeneity. The current meta‐analysis revealed a significant difference in the incidence of RBO after EUS‐HGS and EUS‐CDS; however, this was not the case for TRBO. This might be due to patient survival. In many cases, biliary obstruction was caused by advanced pancreatobiliary cancer; therefore, patient survival time was short. Indeed, most patients died before the onset of RBO. This short survival time may have a marked effect on the occurrence of TRBO. By contrast, RBO rates may rather reflect real stent dysfunction. To better compare the TRBO of EUS‐CDS and EUS‐HGS, more prospective studies are needed to reduce the level of heterogeneity.

We also found that the procedure time for EUS‐CDS was shorter than that for EUS‐HGS. This result was the same as that reported in a previous meta‐analysis.^30^ This is probably because extrahepatic biliary access is easier for EUS‐CDS than that for EUS‐HGS^33^; also, manipulating the guide wire for EUS‐HGS is more difficult than for EUS‐CDS.^11^ In general, EUS‐HGS requires a greater number of accessory changes than EUS‐CDS.^34^

This meta‐analysis has several limitations. First, the articles selected did not use uniform definitions of clinical success, AE, and TRBO. In the future, only articles that use the same definitions, and provide sufficient data required for meta‐analyses, should be selected. Second, the high dispersion and heterogeneity upon visual analysis of the plot for TRBO might have affected the interpretation of the data and the conclusions, although a random effects model (DerSimonian‐Laird method) was used. Third, many different stent types were used, leading to bias with respect to the stent type. Fourth, although this meta‐analysis suggests that EUS‐CDS is superior to EUS‐HGS for AE and RBO, EUS‐CDS is performed mainly for patients who do not have a duodenal obstruction or surgically‐altered anatomy. Therefore, EUS‐HGS can be performed for those who do have a duodenal obstruction or surgically‐altered anatomy. EUS‐HGS is more adaptable to patients with biliary obstruction than EUS‐CDS. Fifth, the cited articles reported different rates of duodenal obstruction, and they included many different primary diseases. These may introduce a strong bias. Finally, the use of chemotherapy was not considered when evaluating RBO and TRBO. To remove potential bias due to patient background, a prospective study is needed to compare detailed data from patients undergoing EUS‐CDS and EUS‐HGS.

## CONCLUSIONS

There were no significant differences between EUS‐CDS and EUS‐HGS with respect to technical success rate, clinical success rate, AE, and TRBO in patients with malignant biliary obstruction. In patients without duodenal obstruction but with biliary obstruction, clinicians may first consider using EUS‐CDS because the RBO rate was lower after EUS‐CDS than after EUS‐HGS. If there are difficulties regarding the use of EUS‐CDS, or the patient has a duodenal obstruction, then EUS‐HGS is an alternative option.

## CONFLICT OF INTEREST STATEMENT

Masayuki Kitano has received honoraria (for lectures) from Olympus, and research funding from Boston Scientific and Zeon Medical. Author Takeshi Ogura is an Associate Editor of DEN Open. The other authors have no conflicts of interest to declare.

## References

[1] Halttunen J , Meisner S , Aabakken L *et al*. Difficult cannulation as defined by a prospective study of Scandinavian Association for Digestive Endoscopy (SADE) in 907 ERCPs. Scand J Gastroenterol 2014; 49: 752–8. 24628493 10.3109/00365521.2014.894120

[2] Bailey AA , Bourke MJ , Williams SJ *et al*. A prospective randomized trial of cannulation technique in ERCP: Effects on technical success and post‐ERCP pancreatitis. Endoscopy 2008; 40: 296–301. 18389448 10.1055/s-2007-995566

[3] Morales SJ , Sampath K , Gardner TB . A review of prevention of post‐ERCP pancreatitis. Gastroenterol Hepatol 2018; 14: 286–92. PMC6034611 29991936

[4] Cho DH , Lee SS , Oh D *et al*. Long‐term outcomes of a newly developed hybrid metal stent for EUS‐guided biliary drainage (with videos). Gastrointest Endosc 2017; 85: 1067–75. 27650270 10.1016/j.gie.2016.09.010

[5] Park DH , Jang JW , Lee SS , Seo DW , Lee SK , Kim MH . EUS‐guided biliary drainage with transluminal stenting after failed ERCP: Predictors of adverse events and long‐term results. Gastrointest Endosc 2011; 74: 1276–84. 21963067 10.1016/j.gie.2011.07.054

[6] Kim TH , Kim SH , Oh HJ , Sohn YW , Lee SO . Endoscopic ultrasound‐guided biliary drainage with placement of a fully covered metal stent for malignant biliary obstruction. World J Gastroenterol 2012; 18: 2526–32. 22654450 10.3748/wjg.v18.i20.2526 PMC3360451

[7] Celso AJ , Lopes CV , Kemp R , dos Santos JS . Different options of endosonography‐guided biliary drainage after endoscopic retrograde cholangio‐pancreatography failure. World J of Gastrointest Endosc 2018; 10: 99–108. 29774089 10.4253/wjge.v10.i5.99 PMC5955728

[8] Khashab MA , Messallam A , Penas I *et al*. International multicenter comparative trial of transluminal EUS‐guided biliary drainage via hepatogastrostomy vs. choledochoduodenostomy approaches. Endosc Int Open 2016; 4: 175–81. 10.1055/s-0041-109083 PMC4751013 26878045

[9] Poincloux L , Rouquette O , Buc E *et al*. Endoscopic ultrasound‐guided biliary drainage after failed ERCP: Cumulative experience of 101 procedures at a single center. Endoscopy 2015; 47: 794–801. 25961443 10.1055/s-0034-1391988

[10] Minaga K , Ogura T , Shiomi H *et al*. Comparison of the efficacy and safety of endoscopic ultrasound‐guided choledochoduodenostomy and hepaticogastrostomy for malignant distal biliary obstruction: Multicenter, randomized, clinical trial. Dig Endosc 2019; 31: 575–82. 30908711 10.1111/den.13406

[11] Guo J , Sun S , Liu X , Wang S , Ge N , Wang G . Endoscopic ultrasound‐guided biliary drainage using a fully covered metallic stent after failed endoscopic retrograde cholangiopancreatography. Gastroenterol Res Pract 2016; 2016: 9469472. 27594881 10.1155/2016/9469472 PMC4983388

[12] Park DH , Lee TH , Paik WH *et al*. Feasibility and safety of a novel dedicated device for one‐step EUS‐guided biliary drainage: A randomized trial. J Gastroenterol Hepatol 2015; 30: 1461–6. 26146796 10.1111/jgh.13027

[13] Artifon ELA , Marson FP , Gaidhane M , Kahaleh M , Otoch JP . Hepaticogastrostomy or choledochoduodenostomy for distal malignant biliary obstruction after failed ERCP: Is there any difference? Gastrointestinal Endosc 2015; 81: 950–9. 10.1016/j.gie.2014.09.047 25500330

[14] Ramírez‐Luna MA , Téllez‐Ávila FI , Giovannini M , Valdovinos‐Andraca F , Guerrero‐Hernandez I , Herrera‐Esquivel J . Endoscopic ultrasound‐guided biliodigestive drainage is a good alternative in patients with unresectable cancer. Endoscopy 2011; 43: 826–30. 21833899 10.1055/s-0030-1256406

[15] Paik WH , Lee TH , Park DH *et al*. EUS‐guided biliary drainage versus ERCP for the primary palliation of malignant biliary obstruction: A multicenter randomized clinical trial. Am J Gastroenterol 2018; 113: 987–97. 29961772 10.1038/s41395-018-0122-8

[16] Sassatelli R , Cecinato P , Lupo M *et al*. Endoscopic ultrasound‐guided biliary drainage for malignant biliary obstruction after failed ERCP in low performance status patients. Dig Liver Dis 2020; 52: 57–63. 31409577 10.1016/j.dld.2019.07.009

[17] Kawakubo K , Isayama H , Kato H *et al*. Multicenter retrospective study of endoscopic ultrasound‐guided biliary drainage for malignant biliary obstruction in Japan. J Hepatobiliary Pancreat Sci 2014; 21: 328–34. 24026963 10.1002/jhbp.27

[18] Vila JJ , Pérez‐miranda M , Vazquez‐sequeiros E *et al*. Initial experience with EUS‐guided cholangiopancreatography for biliary and pancreatic duct drainage: A Spanish national survey. Gastrointest Endosc 2012; 76: 1133–41. 23021167 10.1016/j.gie.2012.08.001

[19] Amano M , Ogura T , Onda S *et al*. Prospective clinical study of endoscopic ultrasound‐guided biliary drainage using novel balloon catheter (with video). J Gastroenterol Hepatol 2017; 32: 716–20. 27420770 10.1111/jgh.13489

[20] Ogura T , Chiba Y , Masuda D *et al*. Comparison of the clinical impact of endoscopic ultrasound‐guided choledochoduodenostomy and hepaticogastrostomy for bile duct obstruction with duodenal obstruction. Endoscopy 2016; 48: 156–63. 26382307 10.1055/s-0034-1392859

[21] Tyberg A , Napoleon B , Robles‐Medranda C *et al*. Hepaticogastrostomy versus choledochoduodenostomy: An international multicenter study on their long‐term patency. Endosc Ultrasound 2022; 11: 38–43. 34494590 10.4103/EUS-D-21-00006 PMC8887039

[22] Mantel N , Haenszel W . Statistical aspects of the analysis of data from retrospective studies of disease. J Natl Cancer Inst 1959; 22: 719–48. 13655060

[23] Higgins JP , Thompson SG . Quantifying heterogeneity in a meta‐analysis. Stat Med 2002;21:1539–58. 12111919 10.1002/sim.1186

[24] Higgins JP , Thompson SG , Deeks JJ , Altman DG . Measuring inconsistency in meta‐analyses. BMJ 2003; 327: 557–60. 12958120 10.1136/bmj.327.7414.557 PMC192859

[25] DerSimonian R , Laird N . Meta‐analysis in clinical trials. Control Clin Trials 1986; 7: 177–88. 3802833 10.1016/0197-2456(86)90046-2

[26] Begg CB , Mazumdar M . Operating characteristics of a rank correlation test for publication bias. Biometrics 1994; 50: 1088–101. 7786990

[27] Egger M , Davey Smith G , Schneider M , Minder C . Bias in meta‐analysis detected by a simple, graphical test. BMJ 1997; 315: 629–34. 9310563 10.1136/bmj.315.7109.629 PMC2127453

[28] Kanda Y . Investigation of the freely‐available easy‐to‐use software ‘EZR (Easy R)’ for medical statistics. Bone Marrow Transplant 2013; 48: 452–8. 23208313 10.1038/bmt.2012.244 PMC3590441

[29] Whiting PF , Rutjes AW , Westwood ME *et al*. QUADAS‐2: A revised tool for the quality assessment of diagnostic accuracy studies. Ann Intern Med 2011; 155: 529–36. 22007046 10.7326/0003-4819-155-8-201110180-00009

[30] Mao K , Hu B , Sun F , Wan K . Choledochoduodenostomy versus hepaticogastrostomy in endoscopic ultrasound‐guided drainage for malignant biliary obstruction: A meta‐analysis and systematic review. Surg Laparosc Endosc Percutan Tech 2021; 32: 124–32. 34469370 10.1097/SLE.0000000000000992 PMC8812416

[31] Hedjoudje A , Sportes A , Grabar S *et al*. Outcomes of endoscopic ultrasound‐guided biliary drainage: A systematic review and meta‐analysis. United European Gastroenterol J 2019; 7: 60–8. 10.1177/2050640618808147 PMC6374841 30788117

[32] Wang K , Zhu J , Xing L , Wang Y , Jin Z , Li Z . Assessment of efficacy and safety of EUS‐guided biliary drainage: A systematic review. Gastrointest Endosc 2016; 83: 1218–27. 26542374 10.1016/j.gie.2015.10.033

[33] Ogura T , Itoi T . Technical tips and recent development of endoscopic ultrasound‐guided choledochoduodenostomy. DEN Open 2021; 1: e8. 35310149 10.1002/deo2.8 PMC8828248

[34] Paik WH , Park DH . Outcomes and limitations: EUS guided hepaticogastrostomy. Endosc Ultrasound 2019; 8: 44–9. 10.4103/eus.eus_51_19 PMC6896431 31897379

